# Multifaceted roles of arginine in cellular function, signaling, and disease

**DOI:** 10.3389/fchem.2026.1847056

**Published:** 2026-07-24

**Authors:** Talya Mehmood, Muhammad Ali, Mohsin Javed, Rana Muhammad Mateen, Muhammad Nouman, Salah Knani, Reem Alreshidi, Shahid Iqbal

**Affiliations:** 1 Department of Life Sciences, School of Science, University of Management and Technology, Lahore, Pakistan; 2 Department of Chemistry, School of Science, University of Management and Technology, Lahore, Pakistan; 3 Faculty of Medicine, Sirindhorn School of Prosthetics and Orthotics, Siriraj Hospital, Mahidol University, Bangkok, Thailand; 4 Center for Scientific Research and Entrepreneurship, Northern Border University, Arar, Saudi Arabia; 5 Department of Physics, College of Science, Northern Border University, Arar, Saudi Arabia; 6 Department of Chemical and Environmental Engineering, University of Nottingham Ningbo China, Ningbo, China

**Keywords:** arginine sensing, arginine signaling, arginine-rich motif, DNA repair, nuclear localization, vascular tone

## Abstract

Arginine, also known as L-arginine residue, is a dibasic, cationic, conditionally essential amino acid that serves as an integral part of polypeptide chains. Along with its conventional role in protein synthesis, numerous biological processes depend on L-arginine, such as the synthesis of nitric oxide (NO) and regulation of vascular tone, the urea cycle and acid/base homeostasis, nuclear localization, post-transcriptional and post-translational modifications, signal transduction, the transport system, and viral genome assembly. In the physiological context, it plays diverse roles; for example, it correlates with airflow abnormalities in severe asthma, reduces inflammation, promotes muscle regeneration, and improves blood flow through vasodilation. Keeping in view the above, this review is an effort to collate the current knowledge and discuss and appraise the biological and physiological roles of L-arginine as an integral entity within a biological system.

## Graphical Abstract

**Figure:**
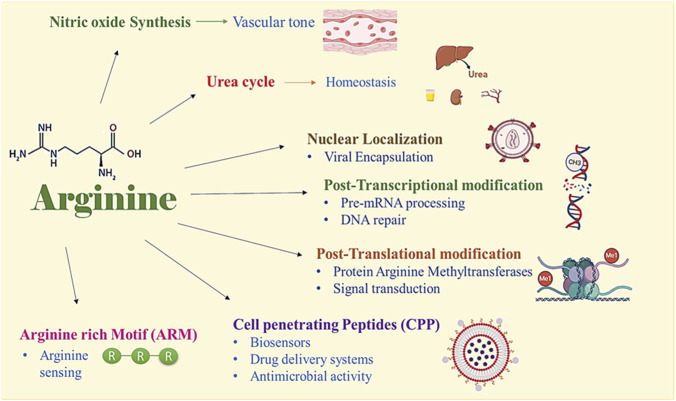
In nitric oxide (NO) synthesis, L-arginine serves as the substrate that helps make nitric oxide, causing vasodilation and maintaining blood pressure. In urea cycle, L-arginine helps in the detoxification of urea and maintenance of homeostasis. Arginine methylation in post-transcriptional modification (PTM) involves pre-messenger RNA processing for proper binding and assembly of survival of motor neuron (SMN) proteins and U–small nuclear ribonucleoprotein (snRNP) machinery. Moreover, arginine methylation is crucial in Krüppel-like factor 4 (KLF4) component for DNA damage repair and encourages genomic stability. In PTM, protein arginine methyltransferase (PRMT) enzymes can methylate R residues of protein, aiding interaction between protein and DNA and influencing either transcriptional activation or repression. Methylated R residues act as attachment sites for particular protein interaction domains, directing signal transduction such as the mTOR pathway. Arginine-rich cell-penetrating peptides are capable of penetrating cell membranes; thus, they can be used in drug delivery systems and biosensors and as potential antimicrobial therapeutics. In viral regulatory systems, proteins like Rev and Tat have arginine-rich motifs that help proteins enter the nucleus and are also useful in packaging materials.

## Introduction

L-arginine is a dibasic and cationic conditionally essential (or semi essential) amino acid. It serves as an integral part of polypeptide chains. It is synthesized/provided through multiple biochemical pathways such as the urea cycle, protein degradation, and dietary intake. Under normal physiological conditions, endogenous synthesis together with dietary intake is generally sufficient to maintain arginine homeostasis; however, during periods of rapid growth, infection, trauma, inflammation, and metabolic stress, external supplementation becomes necessary (Liu et al. 2024). Arginine is therefore considered a conditionally essential amino acid based on this context-dependent requirement. There are six codons encoding arginine in vertebrate genomes—CGU, CGC, CGA, CGG, AGA, and AGG—through arginine aminoacyl-tRNA synthetase. Among these, human mRNAs most commonly use AGG and AGA for encoding arginine. Arginine residue is processed by two iso-acceptor tRNAs having anticodons ICG (decoding CGU, CGC, and CGA) and UCU (decoding AGA, and AGG).

In nitric oxide (NO) synthesis, arginine helps nitric oxide in vasodilation and maintaining blood pressure. In the urea cycle, arginine acts to detoxify urea and maintains homeostasis (Dimeji et al., 2025). Arginine methylation in post-transcriptional modification (PTM) involves pre-messenger RNA processing for proper binding and assembly of survival of motor neuron (SMN) proteins and U–small nuclear ribonucleoprotein (snRNP) machinery. Moreover, in genome stability, arginine methylation is crucial in Krüppel-like factor 4 (KLF4) component for DNA damage repair. In PTM, protein arginine methyltransferase (PRMT) enzymes can methylate arginine in protein, aiding interaction between protein and DNA and influencing either transcriptional activation or repression. Methylated arginines act as attachment sites for particular protein interaction domains, directing signal transduction such as the mTOR pathway. Arginine-rich cell-penetrating peptides are capable of penetrating cell membranes; thus, they can be used in drug delivery systems and biosensors and as potential antimicrobial therapeutics. In viral regulatory systems, proteins like Rev and Tat have arginine-rich motifs that help proteins enter the nucleus and are also useful in packaging materials. Being part of all protein-enriched sources of food, arginine is not likely to be supplemented among healthy individuals.

L-arginine is an effective natural medicinal agent for a variety of disorders, including potent antioxidant characteristics. In clinical trials, L-arginine supplementation is extremely successful as an anti-inflammatory, wound healing, tissue repair, anti-cancer, and neuroprotective approach (Oyovwi and Atere, 2024; Zhang et al., 2026). Conversely, therapeutic depletion of arginine has emerged as a promising strategy in arginine-dependent malignancies. To sum up, along with its role as part of the polypeptide chain, arginine is crucial to sustaining different physiological functions, including amino acid transport mechanisms and nitric oxide-mediated vasodilation, as well as cellular processes such as nuclear regulation, gene expression, and epigenetic modifications (Fung et al., 2025). Furthermore, efforts have been made to bridge theoretical progress in fundamental biochemistry relevance by linking arginine-associated pathways to pathological conditions, including inflammation, muscle growth and regeneration (Hnia et al. 2008), and airway remodeling in severe asthma (Lara et al., 2008). The integrative approach may enable appreciating arginine as a central component of biological systems, where its dysregulation may contribute to disease development and progression.

Moreover, arginine is an important signaling molecule regulating intracellular communication networks. Cellular arginine availability is sensed through nutrient-responsive signaling systems—the mechanistic target of rapamycin (mTOR) complex 1 (mTORC1; Ma et al., 2017), SLC38A9 transporter signaling (Liu et al. 2021), CASTOR1-mediated R residue sensing (Rebsamen et al., 2015), and AMP-dependent metabolic pathways. Despite advances, several important questions are yet to be resolved. For example, the tissue-specific regulation of R-sensing, long-term outcomes of supplementation, and context-dependent effects in inflammatory and neoplastic diseases remain to be completely understood. Addressing these knowledge gaps may facilitate the development of better health strategies.

The present review aims to summarize our current understanding of the multifaceted roles of R residue in cellular function, signaling, and disease, with emphasis on the molecular mechanisms and translational and therapeutic applications. Moreover, opportunities for the future investigation are discussed.

## Methods

The review was performed following PRISMA-2020 (Preferred Reporting Items for Systematic Reviews and Meta-Analyses) guidelines.

## Literature

Literature search was performed using PubMed and Google Scholar databases. Studies published between January 2010 and December 2025 were primarily considered, and pioneering studies published earlier were also included.

## Search terms

The search strategy combined Medical Subject Heading terms and keywords, including L-arginine, arginine metabolism or arginine signaling and nitric oxide, mTOR, immune response, cell proliferation, vascular function, and disease or therapy.

### Study criteria

Peer-reviewed original research articles, patent files, systematic reviews, animal studies, and clinical trials published in English were considered for the study. Studies lacking mechanistic or clinical relevance were excluded.

### Nitric oxide production and vascular tone

Nitric oxide is an important signaling molecule and is available in three isoforms, including neuronal NO, which helps maintain vascular tone and overall cardiovascular health. It regulates blood vessel relaxation through vasodilation, ensuring adequate blood flow, normal blood pressure, and oxygen delivery to tissues. It prevents excessive vascular constriction, lowers systemic resistance, and protects against hypertension (Table 1).

**TABLE 1:** Attributed functions of L-arginine in physiology and pathology.

SN	Function	Reference
1	Provides pre- and post-workout support	Kaewsatuan et al. (2024)
2	Improves blood flow through vasodilation	
3	Supports muscle build-up	
4	Treats erectile dysfunction	Barassi et al. (2017)
5	Improves radiation treatment efficacy in patients with cancer with brain metastases	Marullo et al. (2021)
6	Arginine depletion through arginine decarboxylase may be effective against colorectal cancer as noted in an *in vitro* cell line study	Wei et al. (2023)
7	Reduces symptoms of angina	Excellent review by Cylwik et al. (2005)
8	Lowers blood pressure in people with hypertension	
9	Arginine bioavailability is associated with airflow abnormalities in patients with severe asthma	Lara et al. (2008)
10	Decreases inflammation and promotes muscle regeneration	Hnia et al. (2008)
11	May cause hyperargininemia, which is excessive levels of arginine in the blood owing to defects in the urea cycle	Smith and Garg (2017)
12	Arginine insufficiency activates stress kinase pathway, impairing T lymphocyte function, and inhibits MAPK pathway necessary for macrophage production of cytokines against bacterial encoded endotoxin/lipopolysaccharide	Morris (2012)

**FIGURE 1:**
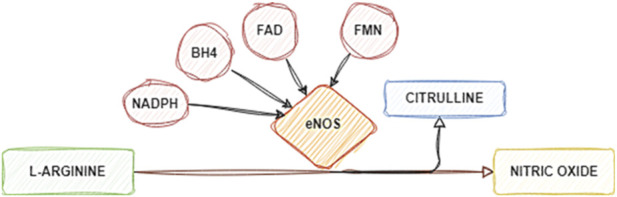
Enzymatic NO production. In a series of redox reactions, L-arginine is degraded to nitric oxide and citrulline by the action of the eNOS enzyme along with the cofactors (FAD, BH4, FMN, and heme) in the presence of NADPH and oxygen. FAD, flavin adenine dinucleotide; FMN, flavin mononucleotide; eNOS, endothelial nitric oxide synthase; BH4, tetrahydrobiopterin; and NADPH, nicotinamide adenine dinucleotide phosphate

Endothelial cells use arginine as a substrate to produce NO, which in turn triggers the control of vascular tone and general cardiovascular homeostasis (Khan et al. 2018). The reaction occurs when the enzyme NO synthase (NOS) produces NO from arginine. This takes place when electrons move from nicotinamide adenine dinucleotide phosphate (NADPH) to heme in the N-terminal oxygenase domain. Consequently, the C-terminal reductase domain’s flavin adenine dinucleotide (FAD) and flavin mononucleotide (FMN) oxidize the arginine substrate to produce citrulline and NO (Figure 1; Gambardella et al., 2020).

Furthermore, NO diffuses into vascular smooth muscle cells and activates soluble guanylate cyclase, increasing cyclic guanosine monophosphate (cGMP) levels and promoting vasodilation. Impaired arginine availability, increased arginase activity, oxidative stress, and reduced NO bioavailability contribute to endothelial dysfunction further associated with hypertension, atherosclerosis, and other cardiovascular disorders (Carlström et al., 2024). In addition to vascular signaling, L-arginine participates in ammonia detoxification.

### Urea cycle and acid/base homeostasis

**FIGURE 2:**
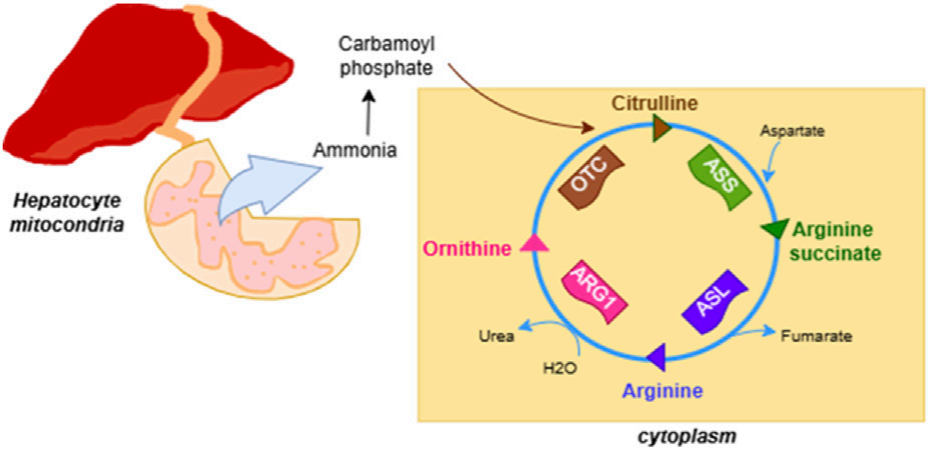
Urea cycle. The urea cycle initiates in hepatocyte (liver cells) mitochondria, where toxic ammonia (i.e., NH_3_ produced during amino acid/protein metabolism) forms carbamoyl phosphate. It then couples with ornithine carboxylase (OTC), converts into citrulline, and exits mitochondria to enter the cytoplasm. Here, citrulline interacts with aspartate and produces arginine succinate through argininosuccinate synthetase (ASS). The enzyme argininosuccinate lyase (ASL) then breaks down the arginine succinate to form arginine and fumarate. Finally, arginase 1 (ARG1) enzyme hydrolyzes arginine-releasing urea (to be secreted via urine through the kidneys), thereby regenerating ornithine.

During enzymatic reaction in the urea cycle, arginine is converted into regenerative ornithine and urea by the enzyme arginase, a manganese metalloenzyme. This step is crucial, as arginine helps in ammonia detoxification through urea production (Figure 2). Arginase is available in two isoforms, arginase I and arginase II. In humans, arginase I is produced by the ARG1 gene and is predominantly expressed in the liver. However, arginase II is expressed by the ARG2 gene and produced in other tissues (Mew et al., 2015). Any mutation in the expression of the ARG1 gene (mostly in conserved regions) leads to decreased activity or deficiency of arginase 1 enzyme, which causes arginine accumulation—avoiding the breakdown of arginine into urea and ornithine—increasing neuronal toxicity and ultimately leading to death (Gambardella et al., 2020).

Along with its role in urea cycle, arginine regulates cellular functions through epigenetic mechanisms.

### Post-transcriptional modification

Arginine methylation, a PTM, is involved in the processing of pre-mRNA, including alternative splicing. In mammalian cells, the assembly of each spliceosome snRNP particle initiates in the cytoplasm. This assembly process involves the formation of a seven-membered ring composed of core Sm proteins, namely, SmB/B′, SmD1, SmD2, SmD3, SmE, SmF, and SmG, in conjunction with a newly exported small nuclear RNA (snRNA) such as U1, U2, U4, or U5 (Yong et al., 2004). To sum up, Sm proteins are a family of small proteins that play crucial role in RNA metabolism, i.e., splicing of nuclear RNA to mRNA.

The SMN protein, essential for cytoplasmic U-snRNP assembly, selectively binds to the dimethyl Arg-modified Arg-Gly repeat domains of the Sm proteins SmD1 and SmD3. This may show the functional significance of methylation modification in U-snRNP biogenesis. The Luhrmann group (Brahms et al., 2001) extended the functional importance of symmetric dimethyl-arginine (sDMA) in SmB/B′ and the Sm-like protein LSm4 and showed that the four Sm/Sm-like proteins (SmD1, SmD3, SmB/B′, and LSm4) interact better with the SMN protein when they are symmetrically dimethylated (Staněk and Neugebauer, 2006). An enzyme family called protein arginine N-methyl transferases (PRMT) is responsible for arginine methylation in histone proteins. A mutation in the huntingtin (htt) gene causes Huntington’s disease, a neurological condition. In Huntington’s disease, altered arginine methylation patterns caused by compromised type 1 PRMT/HTT relationship avoid RNA-binding proteins and mis-splicing of pre-mRNA (Ratovitski et al., 2023). Moreover, abnormal arginine methylation by PRMT5 in spinal muscular atrophy causes exon 7 reduction in the SMN2 gene (Kordala et al., 2023).

### DNA damage repair

KLF4 is a crucial component in the precise molecular signals that cells use to determine whether to repair DNA damage or pursue other survival strategies. Arginine methylation within the unstructured C-terminal region (R374, R376, and R377) may affect the function of KLF4. Similarly, studies suggest that methyl-arginine deposition in the N-terminal region of KLF4 might block or undergo a conformational shift. This mechanism could counteract ubiquitination while maintaining the transcriptional activity of KLF4 (Lorton and Shechter, 2019). Upon sensing DNA damage, PRMT5 methylates KLF4, thereby inactivating KLF4. This may encourage DNA repair, highlighting the role of arginine methylation and ubiquitin in triggering the DNA damage response.

### Post-translational modifications

Arginine methylation is important in the regulation of various biological processes in mammals, e.g., regulation of gene expression, signal transduction including Arg-sensing, and DNA damage repair.

## Gene expression

Methylated R residues can influence the specific interaction of proteins and DNA, thereby affecting transcriptional activation or repression. PRMTs transfer the methyl group from S-adenosylmethionine to the nitrogen atoms of an R residue within the H3 histone protein. This may lead to the formation of mono- or dimethylated R residues in histone H3, contextually activating or repressing gene transcription (Ma et al., 2023). For instance, transcriptional activation is linked to the asymmetric dimethylation of H3R17 and H3R26. Methylated R residues serve as recognition sites for proteins containing specialized protein domains known as “reader” domains. These reader proteins can interpret the histone code and recruit other chromatin-modifying enzymes or transcriptional regulators to the site of methylation, further influencing gene expression (Ma et al., 2023). Contrary to the above, the symmetric dimethylation catalyzed by PRMT5 on histone H4R3 and H3R8 is recognized as a repressive mark associated with gene expression inhibition (Liu et al., 2020). Moreover, dysfunctional regulation of arginine methylation via either PRMT overexpression or hyperactivity induces cancer growth and therapeutic resistance. For instance, in a recent study, it was identified that PRMT3 is involved in oxaliplatin (drug) resistance for the treatment of hepatocellular carcinoma (HCC) due to the methylation of IGF2BP1 at R452 (Shi et al., 2023). Likewise, elevated expression of PRMT3 in gemcitabine (drug)-resistant pancreatic cancer cells was found to cause gemcitabine-associated pancreatic cancer treatment to be unresponsive. In another study PRMT4, PRMT5, and PTMT7 were raised in breast, colorectal, and prostate cancer cells, and they corresponded with elevated levels of hnRNPA1 arginine methylation and abnormal alternative splicing (Li et al., 2021).

PRMT2 is highly expressed in colorectal cancer (CRC) and negatively correlates with tumor malignancy (e.g., less expression more aggressive cancer). PRMT2 promotes CRC malignant phenotypes by activating the Wnt/β-catenin signaling. At least, in CRC, PRMT2 has context-dependent roles. PRMT2 promotes M2 macrophage polarization (supports tumor-supportive microenvironment) and suppresses CD4^+^/CD8^+^ T-cell infiltration (promotes tumor escape from immune surveillance) (Zou et al., 2025). Arginine deprivation is considered a strategy to treat cancers. Many cancers, such as pancreatic, colorectal, and hepatocellular carcinoma, are dependent on external arginine sources. Therapies considering arginine depletion may inhibit tumor growth (Feng et al., 2025).

### Signal transduction

**FIGURE 3:**
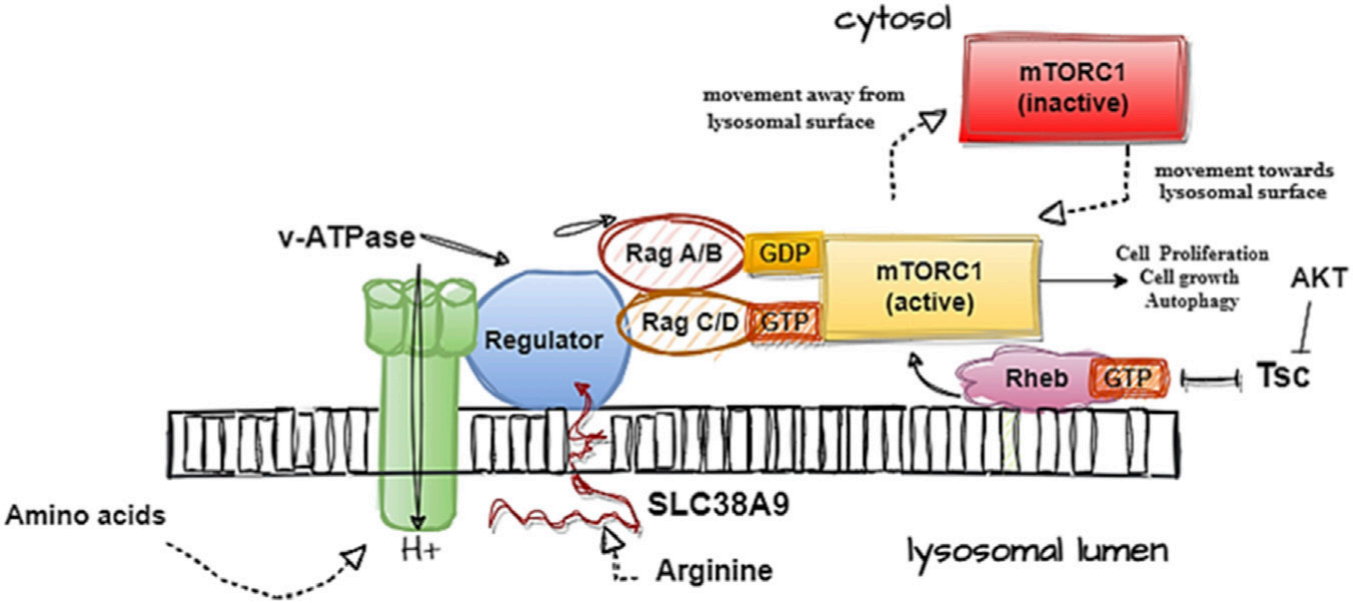
Arginine-dependent mTOR complex activation. Sensing the presence of arginine, the transmembrane protein SLC38A9, shown in the graphic, interacts with the Rag GTPases family of enzymes to activate the mTORC1 growth-regulating metabolic pathway: ATPase, vacuolar ATPase; SLC38A9; Rag A/B, Ras-related binding A/B; RAG C/D, Ras-related binding C/D; GDP, guanosine diphosphate; GTP, guanosine triphosphate; Rheb, Ras homolog enriched in the brain; TSC, tuberous sclerosis complex; mTOR, mammalian (or mechanistic) target of rapamycin; AKT, AK mouse strain transforming (also known as protein kinase B).

Methylated R residues within proteins can act as docking sites for specific protein interaction domains, influencing signaling pathways. There are at least two ways that arginine activates mTOR, i.e., through Rheb or Rag (Figure 3; Liu et al., 2021). First, mTOR may be activated upon growth factor stimulation by deactivating the tuberous sclerosis complex (TSC), a negative regulator of mTOR, via the mitogen-activated protein kinase (MAPK) or phosphoinositol 3-kinase (PI3K) pathways. Rheb, a brain-enriched Ras homolog, is activated when TSC is inhibited. This activates mTORC1. Second, arginine activates mTOR by interfering with the interaction between TSC and mTORC1. Regulator/Rag GTPase, which is necessary to attract the mTORC1 complex to the lysosomal surface, is activated in lysosomes when arginine interacts with SLC38A9 (lysosome transmembrane protein responsible for amino acid arginine transport) and v-ATPase, upstream regulators of mTORC1 (Figure 3; Takahara et al., 2020). Arginine interferes with the CASTOR complex, a negative regulator of Rag A, in the cytoplasm by interacting with CASTOR1, the cytoplasmic arginine sensor for the mTORC1 subunit. This activates RAPTOR (regulatory), a Rag A component of mTORC1. mTOR-related protein redistributes mTORC1 to lysosomes (Ma et al., 2017; Rebsamen et al., 2015).

### Nuclear localization

**FIGURE 4:**
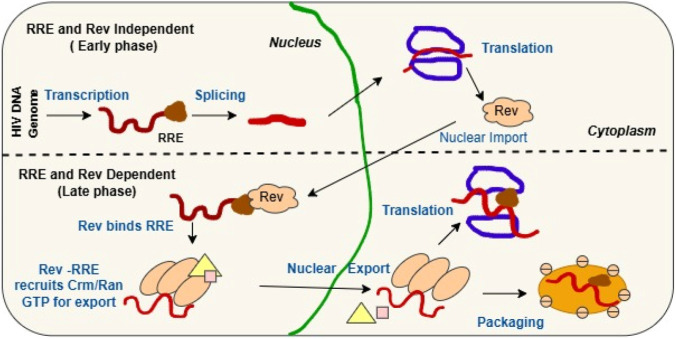
Role of Rev protein NLS pathway in HIV-1. In the early stages of the HIV lifecycle, Rev protein, which is required for the generation of viral structural proteins, is transcribed in the nucleus via completely spliced Rev mRNA and transported to the cytoplasm for translation. Rev arginine-rich NLS binds to importin and is transported back to the nucleus. As the lifecycle progresses, Rev binds to the RRE, which contains large unspliced or partially spliced nuclear RNAs, and interacts with the cellular nuclear export machinery. This process is rendered possible by Rev’s NES. RRE-containing RNAs avoid splicing and are transported to the cytoplasm, where they are either translated into viral proteins or packaged into virions. Rev, regulator of viral protein expression; NLS, nuclear localization signal; NES, nuclear export signal; CRM1, chromosome region maintenance 1; Ran-GTP, Ras-related nuclear protein GTP.

Different cellular and viral regulation systems rely on RNA-binding proteins as a basic component. Viral regulatory systems such as the human immunodeficiency virus (HIV)-1 consist of Rev and Tat proteins with an Arg-rich motif (ARM), a common RNA-binding domain. Rev is also considered a shuttling protein with an ARM that plays a crucial role in controlling the HIV-1 replication cycle (Figure 4). Rev protein comprises both a nuclear localization signal (NLS) and a nuclear export signal (NES). A potential NLS ARM comprises 12 amino acids (35th–46th) in wild-type HIV-1 (Table 2). In the early stages of the HIV lifecycle, the Rev protein crucial for the production of viral structural proteins is transcribed via fully spliced Rev mRNA in the nucleus and exported to the cytoplasm for translation. The Rev protein is transported back into the nucleus through its NLS with an arginine-rich RNA-binding domain, which interacts with cellular importin (alpha/beta) transport machinery.

**TABLE 2:** Arginine-rich cell-penetrating peptide sequences.

Peptide	Sequence (5′-3′)	Significance	Reference
599	GLFEAIEGFIENGWEGMIDGWYGGGGRRRRRRRRRK	An endosome-disruptive fusogenic peptide sequence generated from a synthetic influenza virus and a segment of cationic (D-arginine) residues that penetrate cells	Cantini et al. (2013)
HIV-1 NLS ARM	RQARRNRRRRWR	An arginine-rich binding domain in the NLS of the carrier Rev protein, which particularly interacts with cellular import machinery	Truant and Cullen (1999)
R23F	RKKRRQRRR	An arginine-rich cell-penetrating peptide derived from the ribosomal S1 protein sequence of *S. aureus*	Kravchenko et al. (2022)
R23DI			
R23RI			

As HIV proceeds, Rev attaches to Rev-responsive element (RRE)-containing large unspliced or partially spliced nuclear RNAs and interacts with the cellular nuclear export transport machinery (CRM1, also known as exportin 1, and Ran-GTP). The specific interaction of export processes enables RRE-containing RNAs to avoid splicing and to be transported to the cytoplasm, where they are either translated into viral proteins or packaged into virions (Sherpa and Grice, 2020).

After engaging their respective RNA targets, they become unexpectedly dynamic and can adopt different conformations, such as helical, hairpin, or extended conformations while maintaining the ability to interact with their cognate targets with high affinity and specificity (Ponia et al., 2013). The membrane-translocating properties of arginine-rich motifs have opened avenues for application in therapeutics.

### Arginine-rich cell-penetrating peptides

Peptides rich in arginine are characterized by their unique ability to penetrate cells. Because of their positive charge, they may interact via non-covalent pathways, particularly electrostatic interactions, with negatively charged medicinal molecules and cell membranes. Keeping in view the importance of Arg-rich cell-penetrating peptides, several applications and phenomena have been explored, including biosensors, drug delivery, and antimicrobial activity.

### Biosensors

The increasing focus on Arg-rich peptides in biosensors stems from their notable efficacy in penetrating cell membranes. Among these, the Tat protein has been used as a great delivery tool in biosensors for a long period (Hao et al., 2022). Type 1 HIV Tat protein, released from infected cells, can transport multiple proteins into cells, such as horseradish peroxidase, β-galactosidase, and ovalbumin. Tat, a regulatory protein, contains an arginine-rich protein transduction domain (PTD), the main domain of the Tat protein that helps penetrate the plasma membrane. Scientists have used this feature, and a ratio metric fluorescent zinc biosensor has been developed to enhance the understanding of zinc levels in typical cells. This biosensor was integrated with human carbonic anhydrase and utilized as a sensor transducer to detect cells more accurately.

## Drug delivery

As a drug delivery system, the positively charged Arg-rich peptides, capable of combining with small interfering RNAs (siRNAs), can recruit peptides into targeting cells where they induce silencing of disease-related genes. This is applicable because these peptides hold the capacity to easily cross cell membranes. According to Cantini et al. (2013), one of these peptides, the 599 peptide, demonstrated that the cationic CPP enhanced the bioavailability and intracellular delivery of siRNAs (Table 2). Moreover, research revealed that the 599 peptide fully bound siRNAs by a 50:1 peptide to siRNA molar ratio and facilitated intracellular transport of siRNAs into oral cancer cells with little cytotoxicity. Furthermore, siRNAs combined with the 599 peptide designed to target CIP2A, an oncoprotein overexpressed in human oral squamous cell carcinomas (OSCCs), dramatically reduced the production of CIP2A mRNA and protein. This decreased the *in vitro* invasiveness of cancer cells and anchorage-independent proliferation (Alexander-Bryant et al., 2015; Cantini et al., 2013).

These unique properties further stimulated the development of arginine-based antimicrobial strategies.

### Antimicrobial activity

More recent research has shown that conjugating CPP to AMPs might be helpful for enhancing antibacterial action and selectivity. A few hybrid peptides—R23F, R23DI, and R23EI—were synthesized by Kravchenko et al. (2022). These peptides were derived from the ribosomal S1 protein sequence of *S. aureus* (containing amyloidogenic regions with the propensity to misfold or disrupt normal tissue structure and organ function) and a CPP fragment enriched with R residues (RKKRRQRRR) at their N-termini (Table 2). Upon entry into the bacterial cells, they co-aggregated with functional bacterial proteins. The amyloidogenic site conferred antimicrobial effect. As pathogenic bacteria are becoming increasingly resistant to conventional antibiotics, there is a need to develop new antimicrobial peptides (Kravchenko et al., 2022).

### Arginine-rich motifs

ARM stimulates the nuclear translocation of ORF2, a viral infectious protein, and serves a vital function in the assembly of infectious particles in the HEV lifecycle. The infectious form of ORF2 (i.e., ORF2i) develops a capsid, the outer structure of a virion, while other glycosylated ORF2 proteins (genome-free) may function as immune-evading molecules. Furthermore, ARM may be crucial in determining the fate and actions of ORF2 isoforms (Hervouet et al., 2022). ARM plays a key role in controlling ORF2 nuclear translocation, likely aiding in controlling host antiviral responses during HEV infection.

The motif also controls the signaling ORF2 peptide’s topological duality and efficacy, resulting in either reticular non-infectious glycosylated ORF2 forms or cytosolic infectious ORF2i. In addition, the ARM facilitates the ORF2-host cell membrane connection by acting as a maturation site for furin-mediated glycosylated ORF2. The identification of ORF2 ARM as a unique fundamental regulator of the HEV lifecycle helps explain how viruses strategically condense their genetic information and alter cellular functions (Chen et al., 2021). These interconnected functions together position L-arginine as an important mediator linking metabolism, signaling, and disease.

## Conclusion

L-arginine is a conditionally essential dibasic amino acid that plays diverse physiological roles beyond protein synthesis. It is a key player in NO production, regulating blood flow and vascular tone, and in the urea cycle to detoxify ammonia. The amino acid undergoes post-translational methylation, critical for regulating gene expression, ensuring DNA repair, and processing RNA. This modification influences protein–DNA interactions and signal transduction pathways like the mTOR pathway. For certain viruses, e.g., HEV and HIV, arginine-rich motifs are associated with viral replication and transport of viral proteins into the nucleus. This transport capability is being utilized in drug delivery systems to transport drugs, biosensors, and antimicrobials. Through integration with nutrient-sensing and signaling pathways, arginine influences cellular proliferation, differentiation, immune responses, energy metabolism, and vascular homeostasis. Dysregulation, however, may lead to diverse pathological conditions—cardiovascular diseases, inflammation, metabolic disorders, and cancer. More importantly, arginine’s unique properties are central to maintaining cellular function, genomic stability, and overall physiological health. Strategies involving L-arginine supplementation have demonstrated promising outcomes. However, there is a need for detailed clinical investigations. Future studies combining molecular biology and translational research are expected to refine our understanding and facilitate further development of better therapeutic strategies.

## Future prospects

Despite advances, several important questions need to be addressed—for example, understanding the tissue-specific regulation of arginine sensing and identifying biomarkers related to arginine-based interventions. Further clinical trials are required to determine supplementation regimens, long-term safety, and therapeutic efficacy.
